# Early detection of lysosomal diseases by screening of cases of idiopathic splenomegaly and/or thrombocytopenia with a next‐generation sequencing gene panel

**DOI:** 10.1002/jmd2.12078

**Published:** 2019-12-01

**Authors:** Gloria Muñoz, David García‐Seisdedos, Crina Ciubotariu, Miguel Piris‐Villaespesa, Marta Gandía, Fernando Martín‐Moro, Luis G. Gutiérrez‐Solana, Marta Morado, Javier López‐Jiménez, Antonio Sánchez‐Herranz, Jesús Villarrubia, Francisco J. del Castillo

**Affiliations:** ^1^ UCA de Genómica Traslacional Hospital Universitario Ramón y Cajal, IRYCIS Madrid Spain; ^2^ Servicio de Hematología Hospital Universitario Ramón y Cajal, IRYCIS Madrid Spain; ^3^ Consulta de Neurodegenerativas, Servicio de Neurología Pediátrica Hospital Infantil Universitario Niño Jesús Madrid Spain; ^4^ Centro de Investigación Biomédica en Red de Enfermedades Raras (CIBERER) Madrid Spain; ^5^ Servicio de Hematología Hospital Universitario La Paz Madrid Spain; ^6^ Servicio de Genética Hospital Universitario Ramón y Cajal, IRYCIS Madrid Spain

**Keywords:** genetic screening, lysosomal disease, NGS resequencing panels, splenomegaly, thrombocytopenia

## Abstract

Lysosomal diseases (LD) are a group of about 70 rare hereditary disorders (combined incidence 1:5000) in which diverse lysosomal functions are impaired, impacting multiple organs and systems. The first clinical signs and symptoms are usually unspecific and shared by hundreds of other disorders. Diagnosis of LD traditionally relies on performing specific enzymatic assays, if available, upon clinical suspicion of the disorder. However, the combination of the insidious onset of LD and the lack of awareness on these rare diseases among medical personnel results in undesirable diagnostic delays, with unchecked disease progression, appearance of complications and a worsened prognosis. We tested the usefulness of a next‐generation sequencing‐based gene panel for quick, early detection of LD among cases of idiopathic splenomegaly and/or thrombocytopenia, two of the earliest clinical signs observed in most LD. Our 73‐gene panel interrogated 28 genes for LD, 1 biomarker and 44 genes underlying non‐LD differential diagnoses. Among 38 unrelated patients, we elucidated eight cases (21%), five with LD (GM1 gangliosidosis, Sanfilippo disease A and B, Niemann‐Pick disease B, Gaucher disease) and three with non‐LD conditions. Interestingly, we identified three LD patients harboring pathogenic mutations in two LD genes each, which may result in unusual disease presentations and impact treatment. Turnaround time for panel screening and genetic validation was 1 month. Our results underline the usefulness of resequencing gene panels for quick and cost‐effective screening of LDs and disorders sharing with them early clinical signs.

## 1 INTRODUCTION

Lysosomal diseases (LD) are a group of about 70 rare hereditary disorders in which lysosomal function is altered due to pathogenic mutations that affect the genes encoding lysosomal proteins such as hydrolases, transporters, receptors, enzyme activators and so on. Their combined incidence is 1 in 5000 to 5500,^1^ though individual incidences range from 1:50 000 to 1:250 000. LD are usually inherited following an autosomal recessive pattern, though autosomal dominant and X‐linked forms are also known.

The diagnostic of LD is a challenge. Most patients with LD undergo a “diagnostic odyssey” that may last for years until the correct diagnosis is reached.^2^, ^3^ Two factors contribute to this delay: (a) the insidious onset of many LD, with early symptoms and signs that are unspecific, easy to overlook and common to many other conditions; (b) the rarity of LD, which causes a lack of awareness on these disorders among non‐specialist physicians.^2^ This diagnostic delay is highly undesirable because it may result in unchecked progression of the disorder, application of inappropriate treatments, the onset of complications (including anxiety and stress), a worsened prognosis and the lack of familial counselling.

To reduce this diagnostic delay, universal newborn screening for LD has been implemented in some health systems.^4^, ^5^, ^6^ These newborn screening programs, which rely on high‐throughput enzymatic assays on dried blood spots (DBS), are limited to just a handful of LD with available treatment for which early detection provides demonstrable benefits. For all other LD, the diagnostic strategy is traditionally based, upon clinical suspicion, on assaying enzymatic activities on cultured cells (the gold standard)^7^ or DBS, followed by molecular genetic testing of positive cases to identify the underlying mutations. However, not all LD can be enzymatically assayed and all enzyme assays are available only in specialized laboratories, making such assays impractical to screen every sample for differential diagnosis when the early unspecific signs and symptoms appear. Lack of awareness on LD among first‐line physicians, as indicated, also hampers early suspicion and referral for testing. Thus, most LD are usually underdiagnosed, in particular the late‐onset forms with less severe clinical presentations.

Splenomegaly and thrombocytopenia are the first clinical signs observed in a number of LD and they are among the classical manifestations in most of them, including mucopolysaccharidoses (MPS), mucolipidoses, and sphingolipidoses. The prevalence of these two signs in the general population have been estimated at about 16% for thrombocytopenia^8^ and 2% to 3% for splenomegaly^9^ and they are thus very common findings in the hematology consultation. Indeed, the two signs appear in hundreds of different disorders, both inherited and acquired. Therefore, any diagnostic tool that quickly guides the physician to the right diagnosis would be extremely valuable in clinical practice, in particular if it helps identify infrequent causes for those signs such as any LD. In recent years, next‐generation sequencing (NGS) has demonstrated its usefulness for the differential diagnosis of groups of disorders with high genetic heterogeneity that are difficult to tell apart by non‐genetic means. Here we report on the design, validation and testing of an NGS‐based resequencing panel for genes underlying disorders with splenomegaly or thrombocytopenia among their clinical signs, with specific focus on LD.

## 2 MATERIALS AND METHODS

### 2.1 Subjects

We enrolled 38 unrelated cases of splenomegaly and/or thrombocytopenia of unknown origin referred by hematologists, internal medicine specialists, pediatricians or neurologists from 26 hospitals located all over Spain. This study was approved by the Ethical Committee for Clinical Research of Hospital Universitario Ramón y Cajal. Written informed consent was obtained from all the subjects included in the study or their guardians.

### 2.2 Genetic techniques

DNA was extracted from peripheral blood samples by standard procedures. DNA for panel validation from CEPH 1463 family members was obtained from the Coriell Institute. For genome partition, we used custom SureSelect QXT liquid capture kits (Agilent Technologies) according to the instructions of the manufacturer. Sure Select QXT technology fragments genomic DNA by using a proprietary transposase and subsequently captures selected regions by hybridization of specific RNA probes labeled with biotin. Adaptor‐ and barcode‐tagged gene libraries were deep sequenced in a MiSeq sequencer (Illumina) running in 2x150 bp, paired‐end reading setup. All putative causal variants detected by panel analysis were subsequently verified by Sanger sequencing. For the verification of mutations in those genes of the panel with non‐processed pseudogenes, we performed a gene‐specific, long‐range PCR amplification to generate the template for Sanger sequencing (primer sequences and long‐range PCR conditions available upon request). Additional specific PCR tests were carried out to ascertain the existence of possible whole‐exon deletions whenever suggested by copy‐number variation (CNV) analysis performed with XHMM software (see below).

### 2.3 Data analyses

FASTQ files obtained from the sequencer that passed the quality controls were aligned to the Human Reference Genome (version hg19/GRCh37) with novoalign (http://www.novocraft.com/products/novoalign/). Variants were called with samtools 10 and CNV analysis was performed with xhmm. 11 Variant filtering was carried out on VCF files with Ingenuity Variant Analysis (Qiagen) and all variants detected were assessed by manual inspection of BAM files in the Integrative Genomics Viewer.^12^ Mutation nomenclature is based on cDNA sequences and follows current Human Genome Variation Society rules^13^ as implemented by the mutalyzer 2.0β program (http://mutalyzer.nl). Every case analyzed by means of the panel was discussed in a committee integrated by geneticists and clinical specialists prior to final reporting to the referring clinicians, which followed American College of Medical Genetics (ACMG) guidelines.^14^

## 3 RESULTS

### 3.1 Panel design

Since splenomegaly and/or thrombocytopenia are easily recognizable signs that are among the earliest clinical signs in many LD, we reasoned that screening of cases with idiopathic splenomegaly and/or thrombocytopenia with a gene panel specifically designed to target genes whose mutations underlie such those two signs might lead to early detection of some LD cases. Accordingly, in the splenomegaly and thrombocytopenia panel (STP) we included the 28 genes underlying LD with splenomegaly or thrombocytopenia among their earliest clinical signs. The list includes most sphingolipidoses (*ASAH1*, *GBA*, *GLB1*, *HEXA*, *HEXB*, *GM2A*, *PSAP*, *SMPD1*), MPS (*IDUA*, *SGSH*, *NAGLU*, *HGSNAT*, *GNS*, *ARSB*, *GUSB*) and mucolipidoses (*GNPTAB*, *GNPTG*), as well as glycoproteinoses (*MAN2B1*, *FUCA1*, *NEU1*, *CTSA*), lipid storage diseases (*LIPA*), post‐translational modification defects (*SUMF1*), and lysosomal integral protein disorders (*LAMP2*, *NPC1*, *NPC2*, *SCARB2*, *SLC17A5*).

Given that the STP was meant to provide also a diagnosis for non‐LD cases, we included in our design the following 44 genes underlying non‐LD disorders that also present with splenomegaly and thrombocytopenia: red blood cell membranopathies (*ANK1*, *EPB41*, *EPB42*, *SLC4A1*, *SPTA1*, *SPTB*), hemolytic anemias (*AK1*, *G6PD*, *GPI*, *NT5C3A*, *PIEZO1*, *PKLR*), platelet disorders (*ADAMTS13*, *ANKRD26*, *GATA1*, *GP1BA*, *GP1BB*, *GP9*, *MYH9*, *NBEAL2*, *RBM8A*), erythrocytoses and myeloid malignancies (*BPGM*, *MPL*, *JAK2*, *CALR*, *KIT*, *SH2B3*, *RUNX1*), primary hemophagocytic lymphohistiocytoses (*PRF1*, *STX11*, *STXBP2*, *UNC13D*) and other diverse disorders (*APOA1*, *APOE*, *CLCN7*, *COL2A1*, *FGA*, *HOXA11*, *LYZ*, *SNCA*, *SNX10*, *TCIRG1*, *TNFSF11*, *VWF*). Finally, we also added to the panel the highly polymorphic gene encoding plasma methylumbelliferyl tetra‐N‐acetylchitotetraoside hydrolase (chitotriosidase, *CHIT1*), as this enzyme is commonly used as a biomarker to test the efficiency of many therapies for LD.^15^ For every gene in the STP, the scope included all coding exons, all 5′‐untranslated region exons, all splice sites and any additional sequences in which pathogenic mutations have been observed (eg, deep intronic sites, promoters, etc.), as reported in the ClinVar and COSMIC databases (last accessed on June 30, 2016). In all, the panel targeted 73 genes and 168 different Online Mendelian Inheritance in Man phenotypes. STP interrogated 1248 regions in the human genome, for a total of 248 kb of sequence.

### 3.2 STP validation

To test panel performance, we sequenced a trio from CEPH family 1463 consisting of samples NA12891, NA12892, and NA12878 (father, mother, and daughter, respectively). We compared our results with the complete genomic sequences for these samples available in the 1000 Genomes database (http://www.internationalgenome.org), obtaining values of 99.99% sensitivity, 99.76% specificity, and 97% predictive positive value, at a median depth of 575X. Target sequence of 96.27% had depths greater or equal to 200X, complying with the laboratory standards for NGS of the ACMG.^14^ We also sequenced three blind samples of patients with previous molecular diagnoses and we correctly identified the underlying causative mutations in all of them (Table 1). For all subsequent analyses, we reproduced the conditions used in the validation runs.

**Table 1:** Pathogenic variants detected in the elucidated patients from our cohort

Cases	Sex	Age at referral	Disease diagnosed	MIM #	Gene	Transcript	Variant (cDNA)	Variant (protein)	Variant zygosity	Reference
1	F	9 mo	GM1 gangliosidosis	230500	*GLB1*	NM_000404.3	c.176G>A	p.Arg59His	Homo	16
					*SMPD1*	NM_000543.4	c.1133G>A	p.Arg378His	Hetero	17
2	F	4 y	MPS 3A (Sanfilippo A)	252900	*SGSH*	NM_000199.3	c.707T>C	p.Leu236Pro	Hetero	This work
							c.1027dup	p.Leu343Profs*159	Hetero	18
					*SLC17A5*	NM_012434.4	c.918T>G	p.Tyr306*	Hetero	19
3	F	7 y	MPS 3B (Sanfilippo B)	252920	*NAGLU*	NM_000263.3	c.49_73dup	p.Glu25Glyfs*175	Homo	This work
4	M	31 y	Niemann‐Pick, type B	607616	*SMPD1*	NM_000543.4	c.1829_1831del	p.Arg610del	Homo	20
					*CHIT1*	NM_003465.2	c.1052_1075dup	p.Gly351_Trp358dup	Homo	21
5	F	39 y	Spherocytosis, type 4	612653	*SLC4A1*	NM_000342.3	c.2057C>T	p.Thr686Met	Hetero	rs143131877
6	M	39 y	Gaucher disease	230800	*GBA*	NM_000157.3	c.1226A>G	p.Asn409Ser	Hetero	22
							c.1448T>C	p.Leu483Pro	Hetero	23
			G6PD deficiency	300908	*G6PD*	NM_000402.4	c.70C>T	p.Arg24*	Hemi	This work
7	M	39 y	Type III AD hyperlipoproteinemia	617347	*APOE*	NM_000041.2	c.500_502del	p.Leu167del	Hetero	24
8	M	68 y	Elliptocytosis, type 2	130600	*SPTA1*	NM_003126.2	c.71T>C	p.Ile24Thr	Hetero	25
					*HEXB*	NM_000521.3	c.1250C>T	p.Pro417Leu	Hetero	26
Blind samples	F	28 y	Gaucher disease	230800	*GBA*	NM_000157.3	c.708del	p.Lys237Argfs*17	Hetero	27
							c.1226A>G	p.Asn409Ser	Hetero	22
	M	40 y	Niemann‐Pick, type B	607616	*SMPD1*	NM_000543.4	c.1406A>C	p.Tyr469Ser	Hetero	28
							c.1829_1831del	p.Arg610del	Hetero	20
	M	68 y	Gaucher disease	230800	*GBA*	NM_000157.3	c.1226A>G	p.Asn409Ser	Hetero	22
							c.1448T>C	p.Leu483Pro	Hetero	23
					*CHIT1*	NM_003465.2	c.304G>A	p.Gly102Ser	Homo	29

### 3.3 Cohort analysis

We analyzed by deep sequencing with the STP 38 unrelated patients with splenomegaly (defined in adults as spleen craniocaudal length >120 mm as determined by echography) or thrombocytopenia (defined as platelet count <150 × 10^9^ L^−1^) of unknown origin that were successively referred to our laboratory (Table 2). The cohort consisted of 24 males and 14 females, with a mean age of 35.0 years (range: 1 month to 79 years); 10 of the cases were pediatric (six males and four females with age <14 years). In eight cases of the cohort (21.0%), we identified pathogenic variants in the following genes: *GBA*, *GLB1*, *SMPD1*, *SGSH*, *NAGLU*, *G6PD*, *SLC17A5*, *HEXB*, *SPTA1*, *SLC4A1* and *APOE* (Table 1). In all cases, we verified the existence of the pathogenic mutations detected by the STP by Sanger sequencing. We also used Sanger sequencing to confirm segregation of the variants by analysis of parents, siblings or offspring (when available). Subsequent clinical analyses (Table 2) suggested by the genetic findings confirmed the following diagnoses: GM1 gangliosidosis and Sanfilippo diseases A and B (MPS 3A and 3B) in three pediatric patients, and Gaucher disease, glucose‐6‐phosphate dehydrogenase deficiency, acid sphingomyelinase deficiency (type B Niemann‐Pick disease), type 2 elliptocytosis, type 4 spherocytosis and autosomal‐dominant hyperlipoproteinemia type III in five adult patients. Note that one of the adult patients was diagnosed with two independent conditions (see below). All putative enzyme deficits detected were verified by the appropriate biochemical assays (Table 2). In one case and one of the blind samples used for STP validation, we also detected inactivating *CHIT1* mutations (Table 1) that precluded the use of chitotriosidase as a biomarker for the follow‐up of disease course in those patients. In all cases, complete STP analysis of patients, including Sanger sequencing verification of variants of interest, took about 1 month.

**Table 2:** Clinical features of elucidated patients from our cohort

Case	Sex	Age at referral	Spleen length (mm)	Platelet count (L^−1^)	Other findings reported at referral	Findings reported after genetic screening	Specific enzyme activity assays in blood	Disease diagnosed
1	F	9 mo	68	123 × 10^9^	Coarse facies	Kyphoscoliosis, developmental delay	Beta‐galactosidase: 3 nmol/h/mg protein	GM1 gangliosidosis
2	F	4 y	93	—	Speech regression	GAGs in urine: 35 mg/mmol creatinine; heparan sulfate in urine	—	MPS 3A (Sanfilippo A)
3	F	7 y	98	—	Mild hepatomegaly	GAGs in urine: 38 mg/mmol creatinine; heparan sulfate in urine	N‐acetyl‐alpha‐glucosaminidase: 0.3 nmol/h/mg protein	MPS 3B (Sanfilippo B)
4	M	31 y	220	—	Dyslipidemia	—	Acid sphingomyelinase: 0.1 nmol/h/mg protein	Niemann‐Pick, type B
5	F	39 y	200	100 × 10^9^	Reticulocytes: 240 000 mm^−3^	Spherocytes in peripheral blood smears	—	Spherocytosis, type 4
6	M	39 y	220	118 × 10^9^	Mild hepatomegaly; Reticulocytes: 143 000 mm^−3^	—	Beta‐glucosidase: 0.9 nmol nmol/h/mg protein; glucose‐6‐phosphate dehydrogenase: 2.8 U/mg Hb	Gaucher disease and G6PD deficiency
7	M	39 y	166	74 × 10^9^	—	Sea‐blue histiocytes in bone marrow smears	—	Type III AD hyperlipoproteinemia
8	M	68 y	150	—	Reticulocytes: 210 000 mm^−3^	Elliptocytes in peripheral blood smears	—	Elliptocytosis, type 2

**Figure 1:**
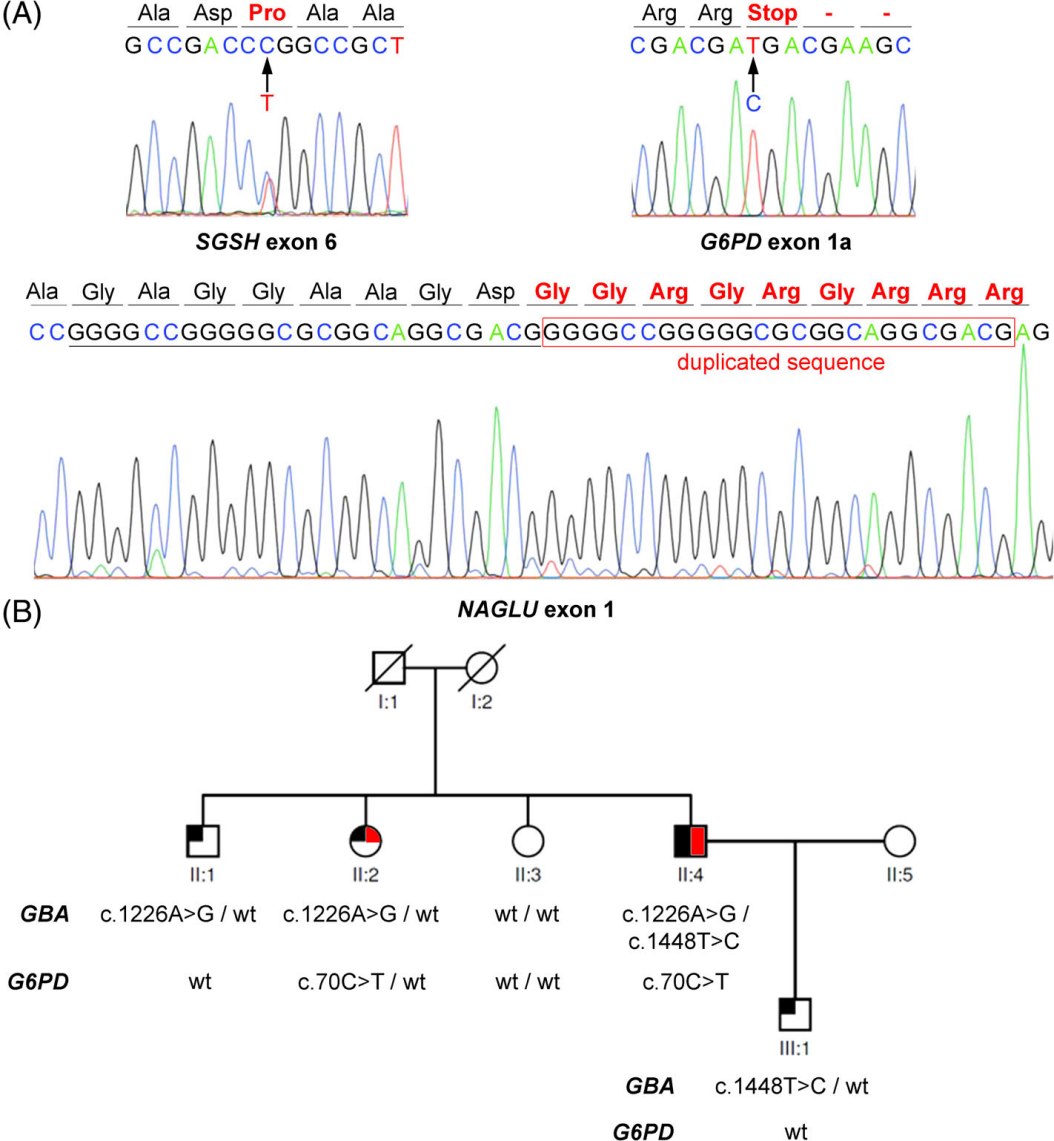
A, Sanger sequence electropherograms of the three novel mutations described in this work: missense c.707T>C (p.Leu236Pro) in *SGSH* exon 6 (heterozygote), nonsense c.70C>T (p.Arg24*) in *G6PD* alternative exon 1 (hemizygote) and frameshift c.49_73dup (p.Glu25Glyfs*175) in *NAGLU* exon 1 (homozygote). B, Pedigree of family PET17 showing segregation of the pathogenic mutations in *GBA* and *G6PD*. The propositus, II:4, is a compound heterozygote for mutations c.1226A>G (p.Asn409Ser) and c.1448T>C (p.Leu483Pro) in *GBA* and a hemizygote for X‐chromosome‐located c.70C>T (p.Arg24*) in *G6PD*. Filled symbol, left half: Gaucher disease; filled symbol, right half: glucose‐6‐phosphate dehydrogenase deficiency

Three of the pathogenic variants detected by the STP had not been reported previously: c.49_73dup (p.Glu25Glyfs*175) in *NAGLU*, c.70C>T (p.Arg24*) in *G6PD* and c.707T>C (p.Leu236Pro) in *SGSH* (Figure 1A). All three variants were evaluated according to the ACMG criteria for interpretation of sequence variants.^30^ The frameshift (c.49_73dup) and nonsense (c.70C>T) variants in *NAGLU* and *G6PD* were classified as “pathogenic” because the two are null variants (criterion PVS1) that are not reported in any of the ExAC (http://exac.broadinstitute.org), 1000 Genomes and gnomAD (https://gnomad.broadinstitute.org) mutation databases (criterion PM2). The missense c.707T>C variant in *SGSH* was deemed “likely pathogenic” because it is located in the sulfatase domain, which is a critical functional domain without benign variation (criterion PM1), it has not been reported in any mutation databases (criterion PM2), we verified that it appears in *trans* with a known pathogenic mutation, c.1027dup, (criterion PM3) and it affects a highly conserved residue (CADD index = 23.3) in which variation is computationally predicted to be deleterious (criterion PP3).

Of note, we identified the c.70C>T (p.Arg24*) nonsense mutation, in hemizygosity, in the X‐linked *G6PD* gene of a male Gaucher patient who was also a compound heterozygote for the pathogenic mutations c.1226A>G (p.Asn409Ser) and c.1448T>C (p.Leu483Pro) (formerly N370S and L444P, respectively) in *GBA*. Assay of glucose‐6‐phosphate dehydrogenase activity in blood indicated pathologic levels of active enzyme (2.8 U/g of hemoglobin (Hb); normal range: 4.6‐13.5 U/g of Hb), confirming the genetic diagnosis of glucose‐6‐phosphate dehydrogenase deficiency, in addition to Gaucher disease. Alternative splicing of *G6PD* transcripts results in the synthesis of up to three enzyme isoforms of different lengths. Since the c.70C>T nonsense variant lies in one of those alternatively spliced exons (exon 1a), the mutation only affects isoform 3 (UniProtKB identifier P11413‐3), which explains the residual activity of glucose‐6‐phosphate dehydrogenase observed in the patient. Segregation analysis in the family confirmed the status of carriers of *G6PD* and/or *GBA* mutations (Figure 1B). Furthermore, we detected that three other patients, an infant with GM1 gangliosidosis, an infant with MPS 3A, and an adult with type 2 elliptocytosis, were also carriers of confirmed pathogenic mutations in the *SMPD1*, *SLC17A5*, and *HEXB* genes, respectively (Table 1).

Interestingly, in a pediatric patient we detected a single, putatively pathogenic variant in the *PRF1* gene: c.11G>A (p.Arg4His). This variant had been previously reported as a cause of dominant aplastic anemia with bone marrow hemophagocytosis (MIM# 609135).^31^ However, further clinical characterization of the patient ruled out aplastic anemia, indicating that the c.11G>A variant is either non‐pathogenic, unlike previously reported, or it is not fully penetrant.

## 4 DISCUSSION

In this work we have tested the usefulness of NGS, gene‐panel‐based genetic screening for disorders underlying two very common clinical signs in the hematology consultation, splenomegaly and thrombocytopenia, for early detection of LD. With the STP, we succeeded in elucidating with minimal delay about one‐fifth (8/38) of the patients that had been referred to our laboratory. Among them, five patients indeed had a LD and we also identified the non‐LD genetic disorders underlying the clinical signs detected in three other patients. Moreover, we identified three LD patients that were carriers of additional pathogenic variants in genes distinct from the one underlying their disease. As these variants result in reduced activities of additional lysosomal enzymes, they should be taken into account in the context of an already malfunctioning organelle. Indeed, carriers of specific *GBA* mutations have increased predisposition to developing Parkinson's disease^32^ and it has been hypothesized that such predisposition may be due to the fact that heterozygosity for a Gaucher mutation could cause a subclinical lysosomal dysfunction that causes a failure in clearing α‐synuclein aggregates. Similarly, the additional heterozygous pathogenic variants detected in these three complex patients may also affect lysosomal function, thus modifying clinical presentation and becoming a source of phenotypic variation that should be considered for follow‐up and treatment. Remarkably, the existence of such variants would probably have been overlooked if the patients had been subjected to the current diagnostic strategy for LD, as second‐tier genetic testing would be exclusively directed at the gene underlying the deficit identified by the enzymatic assays.

Two commonly cited drawbacks of NGS‐based genetic screening strategies are (a) the possibility of missing the causative mutations underlying the disease due to the limitations of the techniques used and (b) what to do in case of identifying variants of uncertain clinical significance (VUS), that is, variants whose pathogenic potential is unclear. The very high sensitivity and specificity (both well over 99%) calculated for the STP indicate that missing causative mutations is rather unlikely. Moreover, we must stress that the STP is intended for quick, early screening of patients *and not as a standalone diagnostic tool*. Indeed, the results obtained with the STP must be confirmed by gold‐standard biochemical assays whenever possible. Thus, patients harboring a VUS or a single confirmed pathogenic allele should be earmarked for further clinical analysis and biochemical testing that would confirm or rule out the suspicion aroused by the genetic data. This point is illustrated here by the case of the patient harboring a single pathogenic mutation in *PRF1*, which turned out to be unrelated to the clinical presentation. Our experience indicates that committee discussion offers the best possibility of elucidating such cases.

As the cost of equipment and consumables for NGS keeps decreasing, whole‐exome sequencing (WES) and whole‐genome sequencing (WGS) are increasingly considered as tools for everyday genetic diagnosis.^33^, ^34^ In WES, sequencing is circumscribed to coding sequences, foregoing the deep intronic and regulatory sequences in which mutations underlying LD have already been identified, regions that we did include in the STP. Furthermore, gene panels, unlike WES, are easily updatable to include additional genes or mutations outside the coding sequence when the advances in research require it. As for WGS, it is certain that because of excellent coverage it will eventually supersede any panel‐based effort, but the large computational workload and time required for analysis and interpretation of WGS data currently makes its use for quick screening impractical.

The diagnostic rate achieved with the STP (21%) might seem low in the light of the results achieved with NGS panels for other diseases (usually greater than 30%). However, two considerations should be borne in mind when analyzing that apparently low yield. Firstly, high‐throughput biochemical analyses elucidate at most 4.6% of patients referred with a clinical suspicion of LD,^35^ implying that the diagnostic yield achieved with the STP (5/38 or 13.2% for LD alone) is a real improvement. Secondly, the STP is indicated for two very common conditions whose cause is usually acquired—indeed, roughly three‐quarters of splenomegaly cases are due to non‐hereditary causes,^9^ whereas the most common causes for thrombocytopenia are chronic alcohol abuse, chronic liver disease, reactions to drugs and immune disorders.^36^ These data suggest that most of the cases that remain unelucidated after STP analysis do not have a genetic cause.

Very recently, Gheldof et al^35^ reported a 14.6% (22/150) diagnostic rate when screening a cohort of patients with clinical suspicion of LD by means of an NGS resequencing panel. Interestingly, when comparing the results of their panel with those of the high‐throughput biochemical analyses that they had previously implemented (4.6% diagnostic yield), they realized that the larger part (9%) of the increase in yield was due to the far greater scope of the NGS panel (51 genes underlying LD) as regards the biochemical assay platform (just 21 enzymes tested). Accordingly, our planned expansion of the STP to include further genes underlying LD should result in higher diagnostic yields.

Motta et al^37^ screened a cohort of 196 patients with splenomegaly and/or thrombocytopenia for early detection of Gaucher disease by analysis of β‐glucosidase activity in DBS. Though the assay used was specific only in 88.5% of cases at the highest sensitivity achieved (88.2%), they succeeded in diagnosing seven patients (3.6%) for the most common LD in Western populations, which validated this approach to early recognition of a LD. Our NGS‐based panel approach can similarly be used by non‐LD expert physicians, with several critical advantages. Firstly, the STP interrogates 28 genes underlying 39 different LD, not just one. Secondly, it simultaneously provides data on 44 other genes underlying differential diagnoses, plus one gene encoding a biomarker commonly used to follow the course of several LD (eg, Gaucher disease, Niemann‐Pick disease, MPS).^15^ Remarkably, other NGS panels used for LD screening do not consider any of these differential diagnoses,^35^, ^38^ which limits their clinical usefulness. Thirdly, the STP helps identify complex patients harboring mutations in several genes, which may result in atypical clinical presentations and must be considered when prescribing therapy. Finally, with both specificity and sensitivity for variant detection over 99%, the STP has the potential for very low rates of false positive and false negative cases if genetic results are discussed in a committee integrating both geneticists and clinicians, as implemented in this work. Altogether, our results show that the STP is a valuable analysis tool for unelucidated cases of splenomegaly and thrombocytopenia, providing a fast and efficient route to diagnosis, even in cases without a prior clinical suspicion of LD or previous biochemical diagnoses.

## CONFLICT OF INTEREST

G.M. received grants and honoraria from Sanofi Genzyme. L.G.G.‐S. received consulting fees from Biomarin, Sanofi Genzyme, Shire Takeda and Ultragenyx. M.M. received grants and honoraria from Sanofi Genzyme. J.V. received grants and honoraria from Sanofi Genzyme and consulting fees from Shire Takeda. F.J.dC. received grants and honoraria from Sanofi Genzyme. All the other authors have no conflict of interest to declare.

## ETHICS APPROVAL

This study (163/16) was approved by the Ethical Committee for Clinical Research of Hospital Universitario Ramón y Cajal on July 16, 2016. All procedures followed were in accordance with the ethical standards of the responsible committee on human experimentation (institutional and national) and with the Helsinki Declaration of 1975, as revised in 2000. Informed consent was obtained from all patients for being included in the study.

## AUTHOR CONTRIBUTIONS

G.M., A.S.‐H., J.V. and F.J.dC. conceived the study. D.G.‐S. and F.J.dC. designed the resequencing panel. G.M., D.G.‐S., C.C. and M.G. performed experiments. M.P.‐V., F.M.‐M., L.G.G.‐S., M.M., J.L.‐J. and J.V. examined patients. G.M., D.G.‐S., M.P.‐V., M.G., J.L.‐J., A.S.‐H., J.V. and F.J.dC. analyzed results. G.M., M.G., J.V. and F.J.dC. wrote the manuscript and completed tables and figures. All authors approved the manuscript.
